# 

**Published:** 1917-04

**Authors:** 


CONTENTS.

ORIGINAL COMMUNICATIONS .................... 1

SELECTIONS AND ABSTRACTS .................... 4

